# The role of cGAS-STING signaling in HPV infection and HPV-related cancers

**DOI:** 10.3389/fimmu.2025.1709613

**Published:** 2025-10-09

**Authors:** Qiugang Zhu, Shiyi Yu

**Affiliations:** Department of Laboratory Medicine, Shangyu People’s Hospital of Shaoxing, Shaoxing University, Shaoxing, China

**Keywords:** HPV, cGAS, STING, infection, cancers, therapeutic target

## Abstract

Human papillomavirus (HPV) is a highly prevalent virus that primarily infects human epithelial cells, resulting in a significant health burden by causing conditions such as anogenital warts, cervical cancers, and head and neck squamous cell carcinoma. Although vaccination has been implemented for cancer prevention, a thorough understanding of anti-HPV immunity remains of critical importance for HPV-related disease management. The cyclic GMP-AMP synthase (cGAS)-stimulator of interferon genes (STING) pathway forms a key signaling cascade within the innate immune system, which is activated by cytosolic DNA and functions through the production of type I interferon (IFN-I). Accumulating evidence indicates a correlation between the cGAS-STING pathway and HPV infection, as well as HPV-related malignancies, suggesting its potential as a promising therapeutic target. This review discusses the role of the cGAS-STING signaling pathway in HPV infection and HPV-related cancers, as well as potential therapeutic strategies that target this pathway.

## 1 Introduction

**Figure 1:**
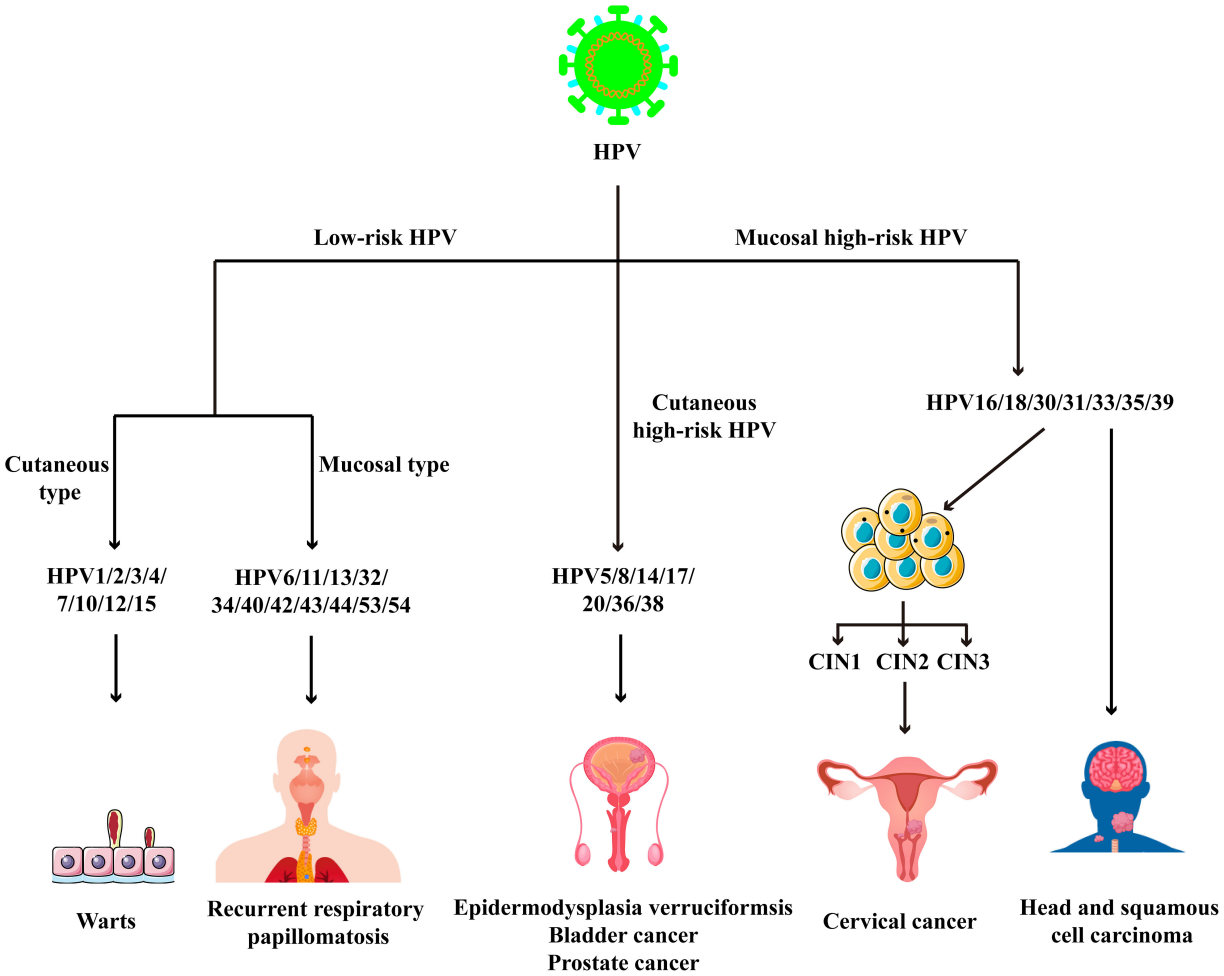
The classification of HPV and HPV-related diseases.

Human papillomavirus (HPV) is a small DNA virus that belongs to the Papillomaviridae family and poses a significant threat to global public health (1–3). Based on the disease outcomes, HPV strains are categorized into low-risk and high-risk types. Low-risk HPV types, such as HPV 6 and 11, are primarily associated with benign lesions, including genital warts and common warts (4). In contrast, high-risk HPV types, including HPV 16 and 18, are strongly linked to the pathogenesis of head and neck squamous cell carcinoma (HNSCC)and cervical cancer (**Figure 1**) (5). The innate immunity serves as the frontline against viruses, employing pattern recognition receptors (PRRs) to detect pathogen-associated molecular patterns (PAMPs). This recognition triggers the production of a wide array of cellular and molecular factors to counteract the viral infection (6, 7). Additionally, innate immunity also plays a pivotal role in HPV-related cancers (8, 9).

The cyclic GMP-AMP synthase-stimulator of interferon genes (cGAS-STING) signaling pathway serves as a crucial axis for type I interferon (IFN-I) induction upon cell-free DNA stimulation (10). Researchers have discovered that the cGAS-STING signaling pathway is closely related to the innate immunity against HPV infection and HPV-related cancers. For instance, HPV E2 attenuates the STING-IFN response to facilitate viral replication, whereas STING activation inhibits cervical cancer growth by augmenting the anti-tumor response (11, 12). The loss of HPV16 E7 restores cGAS-STING responses in HPV-positive oropharyngeal squamous cell carcinomas (13). Also, the cGAS-STING signaling pathway has been considered as a therapeutic target because its activation suppresses tumor growth and overcomes resistance to anti-PD-1 therapy (14, 15).

In this review, we present a concise overview of the cGAS-STING pathway, highlighting its significance in HPV infection and HPV-related cancers. Additionally, we discuss the therapeutic potential of this signaling pathway in the treatment of HPV-related diseases.

## 2 Concise view of cGAS-STING signaling pathway

**Figure 2:**
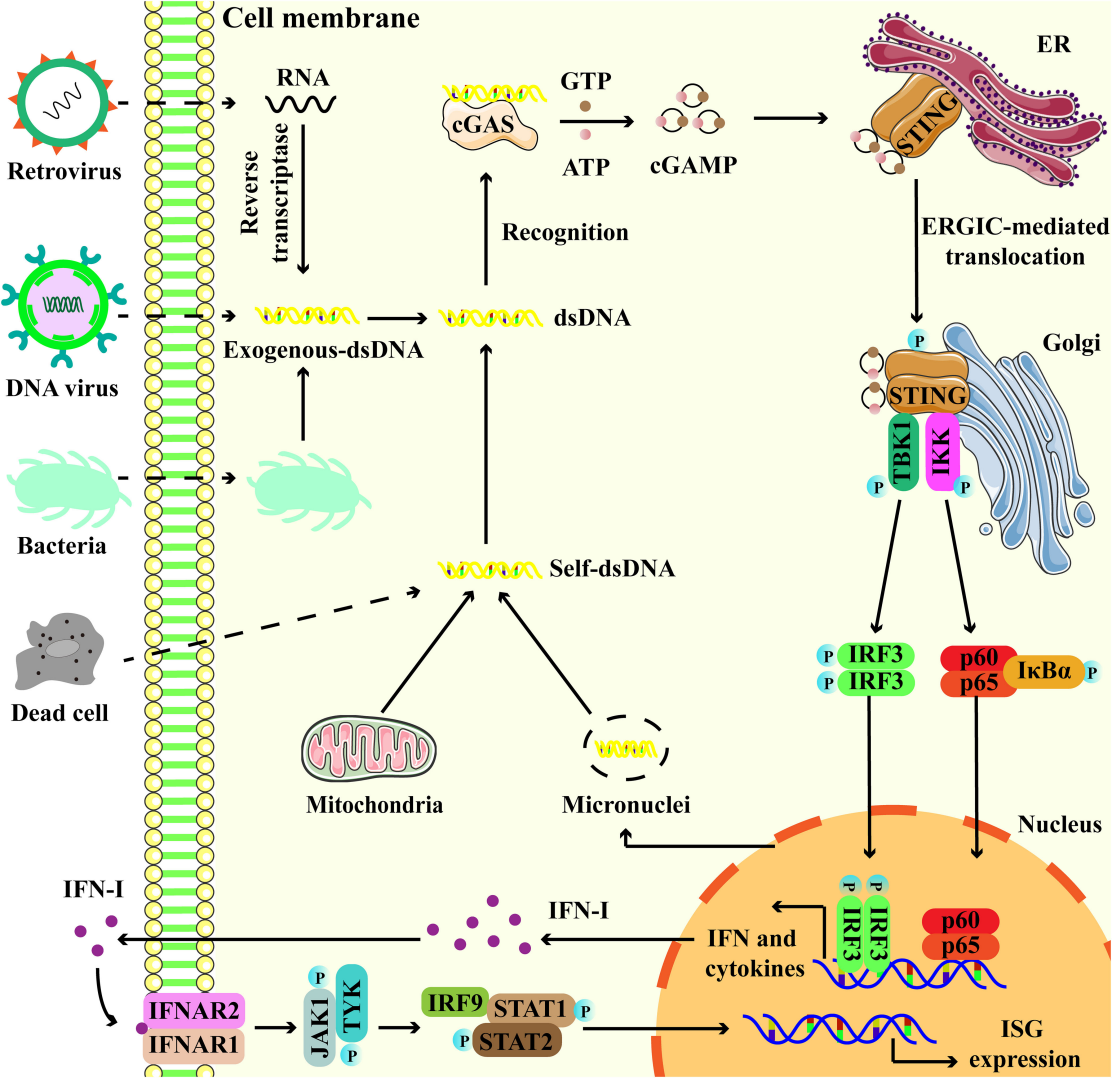
Overview of the cGAS-STING signaling pathway. Exogenous DNA from dead cell, virus and bacteria, and endogenous DNA leakage from micronuclei and mitochondria, interact with cGAS, promoting the activation of cGAS to catalyze the production of 2′,3′-cyclic GMP-AMP (cGAMP) from ATP and GTP. cGAMP binds to endoplasmic reticulum (ER)-located STING, then STING undergoes endoplasmic reticulum (ER)-to-Golgi trafficking via ER-Golgi intermediate compartment (ERGIC). Subsequently, STING serves as a platform to recruit TANK binding kinase 1 (TBK1) and interferon regulatory factor 3 (IRF3), as well as their phosphorylation. Phosphorylated IRF3 translocates to the nucleus and turns on the expression of type I interferons (IFN-I). Besides, STING also interacts and activates IKK, triggering the transcriptional activation of NF-κB. Binding of IFN to IFNAR1-IFNAR2 activates JAK1 and STAT, leading to the induction of interferon-stimulated genes (ISGs).

The cGAS-STING signaling pathway functions as the primary mechanism for cells to sense and respond to double-stranded DNA (dsDNA) in the cytoplasm, thereby establishing a robust innate immune response by inducing the expression of IFN-I (16). In detail, cytoplasmic dsDNA, whether originating from pathogens or cellular components, can be recognized by cGAS. Then, cGAS is activated to catalyze the synthesis of cGAMP from GTP and ATP (17). cGAMP binds to STING, triggering its conformational change and activation. Meanwhile, STING translocates from the endoplasmic reticulum (ER) to the Golgi apparatus through the ER-Golgi intermediate compartment (ERGIC) (18, 19). STING then recruits TANK-binding kinase 1 (TBK1) and interferon regulatory factor 3 (IRF3), resulting in their phosphorylation (20). Phosphorylated IRF3 undergoes dimerization and translocates into the nucleus to induce the expression of IFN-I (**Figure 2**) (20). In addition to the classical pathway, studies have identified non-classical cGAS-STING signaling mechanisms, including cGAS-STING-induced pyroptosis, cGAS-STING-PERK-eIF2α pathway, and STING-induced autophagy (21–23).

The activation of the cGAS-STING signaling pathway is regulated by multiple factors, such as viral components, protein modifications, and other epigenetic mechanisms, thereby influencing a series of physiological and pathological processes (20, 24). The cGAS-STING signaling plays a crucial role in safeguarding the host against viral and bacterial infections (25, 26). In the context of cancer, the cGAS-STING signaling exhibits a dual nature, exerting anti-tumor effects through the induction of IFNs while, paradoxically, fostering tumor progression in certain scenarios via the promotion of chronic inflammation (14, 27).

## 3 cGAS-STING signaling in HPV infection

Unlike other DNA viruses, HPV follows a distinct vesicular trafficking pathway that may interfere with the activation of cGAS-STING DNA-sensing pathway (28). In this study, it was observed that HPV16 pseudovirus evaded cGAS-STING responses during the initial infection, as evidenced by rare transcriptional activation of IFNs and interferon-stimulated genes (ISGs) (28). Furthermore, experimental induction of premature viral penetration of vesicular membranes using membrane-disrupting cationic lipids demonstrated that circumventing natural trafficking pathway of HPV could trigger the activation of the cGAS-STING pathway (28). Therefore, targeting the trafficking process of HPV during the early infection presents a potential strategy to restrict its infection.

E7, originating from HPV, antagonized the cGAS-STING DNA-sensing pathway (**Table 1**) (29). Specifically, it was discovered that the transduction of HPV E7 into primary mouse embryonic fibroblasts inhibited DNA-activated *Ifnb* mRNA expression (29). Further investigations revealed that the LXCXE motif within E7 played a crucial role in antagonizing DNA sensing by interacting with STING (29). Recurrent respiratory papillomatosis (RRP) represents an uncommon benign tumor primarily triggered by HPV6/11 infection in respiratory tract epithelial cells, with the IFN-β response to RRP being subject to regulation by HPV11 E7 (30). A negative correlation was observed between IFN-β levels in localized neoplasms and the HPV load, indicating the disruptive effects of IFN-β on viral replication (30). Moreover, this study demonstrated that HPV11 E7 downregulated IFN-β responses by inhibiting the phosphorylation of STING, as evidenced by the reduction of IFN-β expression in cells expressing HPV11 E7 (30). *In vitro*, macrophages with activated STING exhibited increased production of IFN-β, which subsequently induced the death of HPV-infected epithelial cells and reduced the expression of HPV proteins (30). Interestingly, HPV18 E7 exhibited selective antagonism towards cGAS‐STING-induced NF‐κB activation. In detail, HPV18 E7 interacted with STING, which blocked the nuclear accumulation of p65 and impeded NF‐κB signaling (31). Moreover, E7 inhibition of STING‐triggered NF‐κB activation was related to its HPV pathogenicity (31).

**Table 1:** Effects of HPV components on cGAS-STING signaling.

HPV components	Functions	Effects on cGAS-STING signaling	References
E1, E2, E4	Interacts with STING, TBK1, and IRF3, Suppresses the phosphorylation and nuclear translocation of IRF3	Inhibiting	(12, 32, 33)
E5	Interacts with STING	Inhibiting	(34)
E6	Induces cGAS expression	Sensitizes cells to DNA damage induced apoptosis	(59)
E6, E7	Upregulates topoisomerase I expression	Activates the cGAS-PD-L1 pathway	(41)
E7	Interacts with STING to inhibit downstream signaling	Inhibiting	(13, 29, 30)
	Upregulates the expression of SUV39H1 to suppress the expression of cGAS and STING	Inhibiting	(60)
	Interacts with STING and blocks the nuclear accumulation of p65	Inhibiting cGAS‐STING-induced NF‐κB activation	(31)
	Promotes autophagy-dependent degradation of STING	Inhibiting	(40)

Other HPV proteins, including E1, E2, E4 and E5, have also been demonstrated to downregulate the activation of cGAS-STING pathway (32–35). By mimicking HPV infection, elevated viral titers were detected in HPV16 E2-expressing cells compared to the controls (12). This study demonstrated that E2 interacted with STING, TBK1, and IRF3 to inhibit IRF3 phosphorylation and nuclear translocation, thereby attenuating IFN expression (12). Clinically, STING expression was found to be downregulated in HPV-positive low-grade squamous intraepithelial lesions compared to HPV-negative controls, accompanied by the upregulated HPV E2 transcriptional levels (35). Recently, HPV E1 and HPV E4 have been demonstrated to inhibit IFN responses induced by multiple stimuli, which is mediated by the suppression of RIG-I/MDA5-MAVS, TLR3-TRIF, and cGAS-STING pathways (32, 33). For the cGAS-STING pathway, HPV E1 and E4 interacted with STING, TBK1, and IRF3, leading to the inhibition of both the phosphorylation and nuclear translocation of IRF3 (**Table 1**) (32, 33).

Similar to isoforms of key IFN signaling molecules, the STING isoforms that function as negative regulators of the cGAS-STING pathway have been reported (36–39). STING-ΔC was generated by retention of intron 6, which shared its N-terminal domain with full-length STING but contained a distinct C-terminal sequence (39). Functionally, STING-ΔC interacted with the full-length STING, inhibiting its oligomerization and its assembly with TBK1, which was a crucial component of the STING-TBK1-IRF3 signalosome. This interaction impeded the phosphorylation and nuclear translocation of IRF3, consequently blocking IFN production. Upon HPV16 infection, STING-ΔC downregulated the expression of IFN-β and ISGs (39). STING-ΔN was a shortened STING isoform that lacked 1 to 3 N-terminal transmembrane domains by exon 3 skipping and a new start codon introducing in exon 4, while the C-terminal domain remained intact. STING-ΔN reduced HPV-induced IFN-I and IFN-III production by disrupting the formation of the 2′3′-cGAMP-STING and STING-TBK1 complexes (38).

## 4 cGAS-STING signaling in HPV-related cancers

Increasing evidence has described the role of the cGAS-STING pathway in HPV-related cancers, such as cervical cancer and head and neck squamous cell carcinoma (40–42). The activation of cGAS-STING signaling pathway suppressed cancer progression, which was associated with the apoptosis of cancer cells and infiltration of immune cells within the tumor microenvironment (TME) (14, 43). However, the activation of cGAS-STING signaling can be regulated through different mechanisms.

### 4.1 Cervical cancer

Cervical cancer is the most prevalent cancer type associated with HPV infection, and a growing body of evidence underscores the critical role of the cGAS-STING signaling pathway in cervical cancer. The presence of homozygous variants HAQ/HAQ and R232H/R232H of STING1 was found to correlate with an earlier age at diagnosis and an elevated recurrence rate of cervical cancer, accompanied by poorer survival. Furthermore, patients carrying HAQ/HAQ and R232H/R232H genotypes exhibited a dysfunctional cGAS-STING pathway that failed to stimulate efficient anti-cancer immunity (44). Another study found that advanced-stage cervical cancers were characterized by reduced levels of STING, suggesting that low STING expression could serve as a predictor of worse survival outcomes (45). Moreover, multivariate analyses revealed that STING acted as an independent prognostic indicator for survival in cervical cancer. Interestingly, high STING levels combined with CD103-positive tumor infiltrating lymphocytes were strongly associated with improved prognosis (45). STING-mediated response was inhibited in cervical cancer cells, as evidenced by the reduction of cGAS, STING, and IFN-β expression (11). The knockdown of STING increased the viability and migration of cervical cancer cells, accompanied by the reduction of IFN-β and IL-6 mRNA expression following dsDNA stimulation (11). *In vitro*, ADU-S100 (a STING agonist)-treated cervical cancer cells exhibited reduced cell viability in a dose-dependent manner, accompanied by the upregulation of STING/IFN-β/IL-6 expression (11). *In vivo*, administration of ADU-S100 markedly reduced the volume and weight of xenograft tumors. Concurrently, there was a substantial increase in the number of tumor-infiltrating CD8^+^ T cells and CD103^+^ dendritic cells (11). These findings indicate that the activation of STING enhances the anti-tumor immunity not only through the production of cytokines but also the recruitment of tumor infiltrating lymphocytes.

The presence of PD-L1 downregulates immune response and promotes cancer immune escape (46). IFI16, an interferon-stimulated gene, promoted cervical cancer progression by upregulating PD-L1 expression through the activation of STING-TBK1-NF-κB pathway (47). Compared with HPV-negative cervical cancer cells, the expression levels of IFI16 and PD-L1 were higher in HPV-positive samples. Moreover, IFI16 promoted the expression of PD-L1 and facilitated the oncogenic behaviors of cervical cancer cells (47). Mechanistically, IFI16 activated the STING-TBK1-NF-κB signaling cascade, thereby upregulating PD-L1 expression by promoting the binding between NF-κB and PD-L1 promoter (47). *In vivo*, knockdown of PD-L1 or IFI16 significantly restrained the growth of SiHa-derived tumors (47). A recent study demonstrated that HPV oncoproteins E6 and E7 upregulated topoisomerase I (TOP1) expression to activate the cGAS-PD-L1 pathway, consequently promoting cervical cancer development (41). Specifically, the expression of TOP1 was upregulated in cervical cancer and correlated with poor prognosis. *In vitro*, TOP1 knockdown suppressed cervical cancer cell growth, reduced their migration, and limited their invasion. Moreover, TOP1 inhibition disrupted DNA damage repair and caused cell death. *In vivo*, downregulation of TOP1 markedly suppressed xenograft tumor growth in mice (41).

The downstream genes of STING pathway, such as CCL5, CXCL9, and CXCL10, were found to be closely associated with the prognosis of cervical cancer, as patients with high expression of these genes exhibited improved overall survival and relapse-free survival (15). Moreover, this study demonstrated a correlation between STING downstream genes and immune cell infiltration within the TME of cervical cancer, including CD8^+^ T cells, M1 macrophages, and NK cells (15). The *in vivo* experiment unveiled that MSA-2, a STING agonist, was capable of significantly suppressing the growth of subcutaneous cervical tumors when administered either as a monotherapy or in combination with anti-PD-1 therapy. Furthermore, the combination approach markedly enhanced therapeutic efficacy relative to anti-PD-1 monotherapy (15).

Post-translational modifications (PTMs), including ubiquitination and SUMOylation, play crucial roles in the regulation of cervical cancer (48, 49). It was found that Bcl2-associated athanogene 2 (BAG2) suppressed the progression of cervical cancer by stabilizing STING. Specifically, BAG2 formed a complex with STIP1 homology and U-box-containing protein 1 (STUB1). This complex prevented STUB1 from attaching the K48-linked ubiquitin chains at K338 and K370 of STING, thereby activating the STING-dependent IFN-I pathway to suppress cervical cancer (49). Clinically, there was a positive correlation between BAG2 and STING levels in cervical cancer, with low BAG2 expression strongly linked to advanced disease and poor prognosis (49). A research reported that PIN1 promoted the proliferation and invasion of cervical cancer cells by inhibiting ferroptosis, which was mediated by the inhibition of the cGAS-STING pathway (48). In addition, knockdown of PIN1 significantly downregulated the survival rate of cervical cancer cells. Subsequent investigation suggested that USP34 enhanced the expression of PIN1 via SUMOylation in cervical cancer cells, thereby achieving the tumor-promoting effects (48). However, the role of other PTMs, including glycosylation, acetylation, and palmitoylation, remains to be fully explored preclinically and clinically.

### 4.2 Head and neck cancer

HNSCC represents the predominant histological subtype of head and neck cancers, and HPV16 is the most common subtype associated with HNSCC (50). A previous study revealed that the expression level of STING protein was elevated in HPV^+^ (p16^+^) HNSCC tumor specimens when compared to the HPV^-^ specimens, but only a weak association was observed between high STING expression and improved cancer-specific survival (51). Interestingly, STING activation enhanced cetuximab-mediated NK cell activation and subsequent DC maturation, as indicated by increased expression of CD86, CD83, HLA-DR and PD-L1 on DCs. Moreover, tumor cell STING downregulation diminished cetuximab-mediated NK-DC crosstalk (51). Another study demonstrated that the expression levels of cGAS, TBK1, and IRF3 were similar across all HNSCC cell lines regardless of HPV status, while HPV^−^ cells displayed higher expression of STING compared with low or absent levels in the HPV^+^ cell lines (52). However, both STING-intact HPV^−^ HNSCC cells and STING-overexpressing HPV^+^ HNSCC cell lines exhibited a functional dysregulation of this pathway (52). As opposed to the cell lines, the expression of STING was enhanced in HPV-positive HNSCC patient tissue, and elevated intratumoral STING expression correlated with improved overall survival (52). Another study also indicated that elevated STING expression was associated with improved overall survival in young patients (aged under 60) with HNSCC (40). The different findings regarding STING expression and prognosis in HNSCC among these studies may be attributed to variations in endpoint definition, sample size, and patient status.

Similar to the above findings, it was found that HPV16 E7 was responsible for the inhibition of the cGAS-STING response in HNSCC cells (53). HPV16 E7 shared low homology with HPV18 E7 and employed mechanisms that were distinct from those utilized by HPV18 E7 (40). In detail, HPV16 E7 specifically interacted with NLRX1, resulting in the autophagic degradation of STING. This degradation contributed to the inhibition of cGAS-STING response in HNSCC cells. Moreover, knockdown of HPV16 E7 or NLRX1 deficiency restored the STING signaling and IFN-I induction, as evidenced by increased p-TBK1 expression and downstream molecule expression. *In vivo*, NLRX1-mediated inhibition of anti-tumor immunity was IFN-I-dependent, which was evidenced by the comparable levels of tumor volumes and STING signature genes between groups in *Ifnar1^–/–^* hosts (40).

In HPV-positive oropharyngeal squamous cell carcinomas (OPSCC) cells, knockdown of HPV16 E7 led to a notable restoration of calf thymus DNA-induced IFN-β expression (13). Among patients with squamous cell carcinomas of oral cavity and oropharynx, the proportion of cancers with tumor STING immunoexpression (TSI) was significantly higher in patients with regression of disease than that in patients with progression of disease (54).

In HPV-related carcinogenesis of tongue squamous cell carcinoma (TSCC), it was found that the activation of STING augmented Treg infiltration through the c-jun/CCL22 signaling (27). Interestingly, STING activation promoted the production of immunosuppressive cytokines, such as CCL22, IDO, and IL-10 that played important roles in the development of HPV^+^ TSCC (27). Furthermore, STING activation-induced CCL22 promoted the recruitment of Foxp3^+^ Tregs in HPV^+^ TSCC (27). Mechanistically, c-jun was required for the STING activation-induced CCL22 expression, as silencing of c-jun inhibited the induction of CCL22 at both mRNA and protein levels. Further investigation demonstrated that miR-27 could impair CCL22 production and subsequent Tregs infiltration induced by STING activation, as evidenced by the reduced CCL22 expression and Treg migration *in vitro* (27).

## 5 cGAS-STING signaling: a potential therapeutic target for HPV-related diseases

STING ligands induced rapid regression of murine papillomas, accompanied by enhanced T cell infiltration, IFN-β and TNF-α production (55). Importantly, STING ligands exhibited significantly greater efficacy when compared to Imiquimod (an immunotherapy for papilloma) (55). Activating STING with ligands induced local inflammation and immune responses that specifically targeted HPV-infected and dysplastic cells, potentially resulting in the regression of premalignant lesions and aiding in the control of malignancies (55). Interestingly, this study also found that STING ligands enhanced the survival of mice bearing STING-deficient SCCVII tumors, indicating the therapeutic potential of STING agonists regardless of STING expression by the cancer cells (55). The mutant type of HPV 16 E7 (E7GRG) protein was formulated with 2’-3’cGAMP CDN and/or CpG-C ODN adjuvants to evaluate its immunogenic response and anti-tumor activity (14). In E7GRG + 2’-3’cGAMP+CpG-C-treated tumor-bearing mice, the tumor growth was markedly suppressed, accompanied by the enhanced lymphocyte proliferation response, Th1 cytokine profile, CD8^+^ T-cell responses, granzyme B production, and antibody responses (14).

Studies have demonstrated that STING activation restricted cervical cancer progression, including diABZI, MSA-2, and other STING agonists (15, 43). *In vitro*, a significant downregulation of HPV16/18 E7 protein expression was observed when cervical cancer cells treated with diABZI, accompanied by the reduced cell proliferation. In addition, other STING agonists and radiotherapy (RT) also suppressed its production by activating STING signaling. Interestingly, the STING agonist enhanced the inhibitory effects of radiation on HPV16 E7 protein expression, cell proliferation and clone formation (43). Moreover, this study revealed that activated STING-TBK1 signaling induced the ubiquitin-proteasome degradation of HPV16/18 E7 proteins in cells, which was mediated by E3 ligase HUWE1. Further exploration found that TBK1-mediated phosphorylation of HPV16/18 E7 promoted their ubiquitination and degradation (43). *In vivo*, the STING-TBK1 activation led to a marked suppression of tumor growth, as indicated by reduced tumor volume and tumor weight in tumor-bearing mice (43).

A study revealed the potential of gas-amplified metalloimmunotherapy with dual activation of pyroptosis and the STING pathway for the regulation of immunosuppressive microenvironment in cervical cancer (56). Specifically, PEGylated manganese-doped calcium sulfide nanoparticles (MCSP) were developed, which exhibited the capability to release Ca²^+^, Mn²^+^, and H_2_S in the tumor microenvironment. Besides the calcium overload-induced pyroptosis, H_2_S-induced mitochondrial dysfunction facilitated the release of mtDNA, thereby augmenting the activating effect of Mn²^+^ on the cGAS-STING signaling pathway and consequently activating immunosuppressed dendritic cells (56). *In vivo*, MCSP efficiently triggered the anti-tumor responses, including the upregulation of DC maturation, CD8^+^ T cell percentages, and IFN-γ production. Importantly, the combination of MCSP nanoparticles and PD-1 immunotherapy exhibited synergistic anti-tumor effects and effectively suppressed tumor growth, as evidenced by the pronounced reduction of tumor volume and improved survival rate (56).

Nanoparticles have also been developed to deliver the caffeic acid (CA) for cervical cancer treatment (57). The nanoparticles based on fucoidan (Fu/CA NPs) significantly suppressed the proliferation of cervical cancer HeLa cells, accompanied by the induction of apoptosis. These effects were achieved by the accumulation of reactive oxygen species and mitochondrial damage, which could elicit the activation of cGAS-STING pathway (57). *In vivo*, Fu/CA NPs suppressed solid tumor growth, with even more pronounced anti-tumor effects observed when Fu/CA NPs was combined with cisplatin. The enhanced anti-tumor activity was associated with the activation of the cGAS-STING pathway. Importantly, the combination alleviated cisplatin-induced nephrotoxicity, suggesting the potential of Fu/CA NPs in treating cervical cancer by targeting the cGAS-STING pathway (57). A recent study demonstrated mRNA-encoded STING adjuvant enhanced the efficacy of HPV E6/E7 vaccination in suppressing murine HPV^+^ TC-1 tumor growth, as evidenced by the reduced tumor volume and prolonged survival (58). Moreover, the effects of STING^V155M^ mRNA were dependent on the presence of CD8^+^ T cells, as these effects were abolished when CD8^+^ T cells were depleted. Notably, the production of IFN-γ and TNF-α by CD8^+^ T cells was upregulated when mice were immunized with mRNA-lipid nanoparticles (LNPs) coformulated with HPV E6/E7 and STING^V155M^ (58).

Although these modulators exhibit potential in inhibiting HPV infection and HPV-associated cancers, enhancing their safety and efficacy remains a crucial endeavor. Furthermore, exploring strategies to extend the release and utilization of these modulators (such as the use of nanoparticles or hydrogel systems) represents future directions for clinical research.

## 6 Discussion

Hitherto, it has been clear that innate immunity holds a pivotal position in the development of infectious diseases, autoimmune diseases, and cancers. In the past few years, there has been a significant increase in interest and understanding surrounding the role of cGAS-STING signaling pathway in HPV-related diseases. For instance, HPV18 E7 bound to STING in a region critical for NF-κB activation and then blocked the nuclear accumulation of p65, suppressing the production of inflammatory cytokines (31). Moreover, E5 and E2 have also been reported to inhibit STING-mediated responses, resulting immune evasion (34, 35). Notably, studies pointed out that activation of STING inhibited cervical cancer tumor growth by enhancing the anti-tumor immune response (11). However, some studies confirmed the roles of cGAS-STING signaling in HPV-related cancer development through the upregulation of PD-L1 and expansion of Tregs, indicating the tumor-promoting effects of cGAS-STING activation under certain contexts (11, 47).

Considering the role of the cGAS-STING pathway in HPV infection and HPV-related cancers, targeting the cGAS-STING signaling pathway is promising for their treatments. For example, it has been reported that activation of STING by ADU-S100 reduced the cell viability of cervical cancer cells (11). Notably, STING agonist has been demonstrated to activate the cervical cancer immune microenvironment and overcome anti-PD-1 therapy resistance (15). Moreover, nanoparticles have also been developed for the treatment (57, 58). Due to challenges in medicinal chemistry, rare cGAS-STING modulators have been tested in HPV-related clinical trials. Collectively, future explorations should pay more attention to the following fields: 1) molecular mechanisms regulating the activation of cGAS-STING signaling during HPV infection and HPV-related cancers; 2) the development of novel drugs targeting the cGAS-STING signaling pathway, with emphasis on clinical applications. These efforts may provide new insights into the therapies for HPV infection and HPV-related cancers.
